# Isolation and molecular characterization of Mycobacterium bovis causing pulmonary tuberculosis and epistaxis in a Thoroughbred horse

**DOI:** 10.1186/s12917-016-0813-6

**Published:** 2016-09-02

**Authors:** Tiny Motlatso Hlokwe, David Sutton, Patrick Page, Anita Luise Michel

**Affiliations:** 1 Zoonotic Diseases Section, ARC-Onderstepoort Veterinary Institute, Soutpan Rd., Onderstepoort, 0110 South Africa; 2 Department of Companion Animal Clinical Studies, Faculty of Veterinary Science, University of Pretoria, Soutpan Rd., Onderstepoort, 0110 South Africa; 3 The Weipers Centre Equine Hospital, School of Veterinary Medicine, University of Glasgow, Glasgow, UK; 4 Department of Veterinary Tropical Diseases, Faculty of Veterinary Science, University of Pretoria, Soutpan Rd., Onderstepoort, 0110 South Africa

**Keywords:** Equine, Respiratory, Epistaxis, Zoonosis

## Abstract

**Background:**

Tuberculosis caused by *Mycobacterium bovis* (*M. bovis*) is very uncommon in horses worldwide.

**Case presentation:**

In the current study, an eight-year-old male Thoroughbred in good body condition was admitted to the Equine Clinic at the Onderstepoort Veterinary Academic Hospital in 2005 due to bilateral epistaxis accompanied by coughing. Routine examinations were conducted to determine the cause of the condition. Endoscopic examination revealed the major source of the epistaxis as the trachea, whereas thoracic radiography indicated the presence of a primary pulmonary mass. *M. bovis* was isolated from a broncho-alveolar lavage (BAL) sample collected. The pulmonary mass reduced in size three months later following an oral administration of enrofloxacin (7.5 mg/kg PO SID). Genetic fingerprinting by spoligotyping identified the *M. bovis* isolate as spoligotype SB0868 strain. This *M. bovis* strain type was never described previously in South Africa (SA). This is the first case of *M. bovis* infection in a horse in SA which has been fully documented including clinical findings, isolation and genetic characterisation of the causative pathogen.

**Conclusions:**

This report indicates that horses may contract and harbour *M. bovis* despite their lower susceptibility compared to other domestic animals. It also suggests that the infection may be more easily contained and eliminated from the host.

## Background

*Mycobacterium bovis* is the causative agent of bovine tuberculosis (BTB) in a variety of mammalian hosts. The worldwide status of BTB as a zoonosis is of great concern [1]. Horses are suggested to be more resistant to mycobacterial infections as compared to other livestock animals [2] and tuberculosis incidence in horses is extremely low, especially in countries implementing BTB control programs [3]. Currently, the most common causes of mycobacterial infections in horses are members of the *Mycobacterium avium* complex (MAC) species [4], but under conditions of high infection pressure from co-grazing cattle, *M. bovis* may cause clinical pulmonary infection in horses [5].

Amongst members of the *Mycobacterium tuberculosis* complex (MTBC), *M. bovis* has been more commonly isolated from horses than *M. tuberculosis* [3]. Isolation of *M. bovis* was reported from a cheek lesion of a 9-year-old horse from Nigeria [6]. Acid-fast bacilli were detected on a splenic aspirate from a 10-year-old mare with weight loss in Spain. This mare was euthanized and *M. bovis* infection confirmed by polymerase chain reaction (PCR) and culture of tissue samples. In the cited case, a rapid diagnosis was obtained by use of PCR following ante-mortem detection of acid-fast bacteria [7]. In another report, *M. bovis* was isolated from lymph nodes of a 4-year-old horse in France that had been in close contact with infected cattle, supporting that horses may be more easily infected under conditions of high *M. bovis* infection pressure [5]. Most recently, *M. bovis* infection was confirmed by PCR of tissue samples collected post mortem from a 2-year-old horse with diarrhea and weight loss associated with granulomatous enterocolitis [8]. An antibody response to *M. tuberculosis* antigens was detected in blood samples obtained ante-mortem from a 20-year-old horse with signs of cardiac insufficiency and ventral edema in Switzerland [9]. *M. tuberculosis* pulmonary infection was subsequently confirmed by PCR amplification of DNA extracts from formalin fixed paraffin embedded tissue samples. Generally serological diagnosis of tuberculosis is of limited value in animals and humans and humoral immune responsiveness to MTBC is indicative of progressive tuberculous lesion development [10].

In this report, we describe for the first time, the isolation and molecular characterization of *M. bovis* in a horse in South Africa.

## Case presentation

An 8-year-old, 563-kg Thoroughbred gelding was admitted to the Equine Clinic, Onderstepoort Veterinary Academic Hospital with a primary complaint of bilateral epistaxis, first noted 6 days prior to admission. The epistaxis had increased in severity and frequency, and was accompanied by coughing. The gelding had been purchased for show jumping 1 month previously and transported approximately 1600 km to a new stable yard. No exercise intolerance was reported since purchase.

On presentation the gelding was in good body condition, bright, alert and responsive with good appetite. Body temperature was mildly elevated (38.6 °C; reference range (RR) 37.5–38.5), pulse rate was mildly elevated (46 beats per minute; RR: 28–40) and respiratory rate was normal (16 breaths per minute; RR: <20/min). Mucous membranes were pink and moist with no petechiae. Intermittent, mild epistaxis was evident at both nares. No crackles or wheezes were detected on thoracic auscultation and no spontaneous coughing or coughing on rebreathing examination was recorded. Moderate hypoalbuminemia (22 g/L; RR: 66–83) was present. Serological testing for *Aspergillus* antibodies was negative.

Respiratory endoscopy^1^ revealed petechiae in the medial compartment of the left guttural pouch but no blood. Frank blood was observed in the trachea with hemorrhage originating from a tertiary bronchiole in the left dorsal lung lobe. Trans-endoscopic broncho-alveolar lavage was performed in the region of the hemorrhage and samples submitted for cytology, bacterial and fungal culture. Cytology of the BAL fluid was consistent with pulmonary hemorrhage with chronic inflammation (sero-sanguinous fluid with 42% neutrophils, 23% lymphocytes, and 35 % macrophages with phagocytosed red blood cells). Few free and phagocytosed bacilli were observed. Initial aerobic and anaerobic bacterial and fungal culture results were negative. Standing left lateral thoracic radiographs^2^ revealed a well-marginated soft tissue mass (12 × 15cm) with a dorsal gas cap in the caudodorsal lung field. The fluid line observed in the structure was undulated, indicative of viscous content. A marked peribronchial infiltration with a mild alveolar pattern was observed over the ventral lung field. Thoracic ultrasonography^3^ only revealed pleural surface irregularity of the left lung. Differential diagnoses for the imaging findings included a pulmonary abscess, neoplasia, or fungal granuloma.

Three percutaneous, ultrasound-guided biopsies were collected from the left caudodorsal lung at the site most affected by pleural irregularity. Direct ultrasound-guided biopsy of the pulmonary mass was not possible due to its depth and ultrasound reverberation artefact. Histopathological examination of the lung biopsies showed chronic fibrinous pleurisy of the superficial lung, but was not diagnostic. The owner declined further diagnostic procedures to sample the pulmonary mass and the gelding was discharged with treatment for suspected pulmonary bacterial abscess or fungal granuloma with a course of enrofloxacin^4^ (7.5 mg/kg PO q24h for 8 weeks) and Potassium Iodide(KI^5^)(10 g PO q24h for 6 months), with paddock rest.

Three months later, the horse was re-examined and was found to be in good body condition. The owner reported that there had been no further epistaxis or coughing. Clinical examination was normal and there was no respiratory abnormality detected on auscultation. CBC and fibrinogen results were within reference range. Thoracic radiographs showed moderate reduction in size of the pulmonary mass (9 cm diameter) which was of homogeneous soft tissue opacity, with no gas cap evident. A mild to moderate, focal broncho-interstitial pattern was present caudodorsal to the cardiac silhouette, but was markedly improved compared to previous examination. In view of the reduction in size of the mass, bacterial or fungal granuloma were still considered the most likely differential diagnoses, with neoplasia considered unlikely. The horse was discharged with a further 3-month course of KI. Resumption of ridden walking exercise was recommended with continued monitoring for coughing and epistaxis.

A BAL sample from the initial examination had been subjected to mycobacterial culture according to the laboratory standard operating procedure at the Tuberculosis Laboratory of ARC-Onderstepoort Veterinary Institute. Briefly, the sample was decontaminated for 10 min using 4 % NaOH, centrifuged at 3500 rpm for 10 min and the pellet neutralized with sterile distilled water and centrifuged as before. The resulting pellet was inoculated onto Löwenstein-Jensen media slopes, supplemented with glycerol and pyruvate known to promote growth of *Mycobacterium bovis (M. bovis),* and incubated at 37 °C. Mycobacterial identification of acid-fast colonies was done as previously described [11]. In brief, the mycobacterial isolates were confirmed as members of the *Mycobacterium tuberculosis* complex (MTBC) by using PCR amplification of the MTBC specific 372 bp product and as *M. bovis* by amplification of the 206 bp product within the RD9 region of difference in MTBC organisms. These results were available only 4 months after initial presentation and treatment of the horse, and by which time radiographic evaluation had indicated reduction in size of the pulmonary mass. The zoonotic implications of this condition were discussed with the owner as soon as mycobacterial involvement was confirmed but further testing and isolation could not be enforced.

Spoligotyping, spacer oligonucleotide typing, which detects variability in the direct repeat region in the DNA of MTBC species including *M. bovis*, and VNTR typing based on variation in the number of tandem repeat sequences in the chromosomes of MTBC species, were used for retrospective molecular characterization of the isolate as previously described [12, 13]. Spoligotyping also confirmed the isolates as *M. bovis*, based on the lack of spacers 3, 9, 16, 39–43. Additional spacers absent were 4, 5 and 13. This pattern was consistent with spoligotype SB0868 reported in the international *M. bovis* database (www.mbovis.org). VNTR typing based on a 13 loci VNTR panel was performed as described previously [13], and revealed a unique VNTR profile (6425346436553) which has not been detected in South Africa before.

Six years after initial examination the then 15-year-old gelding was admitted by a new owner for colic and standing, laparoscope-assisted surgery for left dorsal displacement of the large colon was performed. No link was made to the previous admission due to the new owner being unaware of the previous diagnosis. No respiratory tract abnormalities were reported or detected, however, and the horse made an uneventful recovery from surgery and was discharged. The gelding was again re-examined 2 years later as a routine follow-up in this study. He had been in full work with no report of epistaxis or coughing. Physical examination disclosed no abnormalities. Thoracic ultrasonography^6^ revealed mild, bilateral pleural surface irregularity of the ventral lung fields, considered incidental. CBC and serum amyloid A were within reference range. The owner declined further diagnostics and respiratory tract sampling to support bacteriological status or cure.

The present report describes the unusual clinical presentation of epistaxis in a horse with a pulmonary mass and ante-mortem detection of a *M. bovis* strain not previously identified in South Africa. Epistaxis as a presenting clinical sign has not previously been reported with tuberculosis in horses. More common causes of epistaxis in horses, that were excluded in this case, include trauma, exercise induced pulmonary hemorrhage, ethmoid hematoma, guttural pouch mycosis and coagulopathy [14]. The case reported was treated with a 2-month course of fluoroquinolone antimicrobial, which was associated with resolution of clinical signs and concurrent reduction in size of the pulmonary mass. In human medicine, fluoroquinolones are classified as second line drugs for the treatment of tuberculosis [15]. The bactericidal activity of fluoroquinolones against *M. tuberculosis* is important to provide a rapid reduction in infective load and to sterilize the infection [16]. Furthermore, use of moxifloxacin fluoroquinolone in a combination treatment regimen with rifampin and pyrazinamide shortened the time to negative lung cultures, compared to a standard regimen in a murine model [17]. Whilst fluoroquinolones are currently regarded as protected antimicrobials in horses [18], the murine efficacy results may explain the positive clinical response to empirical therapy in the equine case reported. Definitive diagnosis of equine tuberculosis generally relies on post-mortem examination with histopathology, culture and PCR of tissue samples [9].

Infection with *M. bovis* in horses appears to occur mostly by ingestion and the most common site of infection is the gastro-intestinal tract [3]. This is considered unlikely in the reported case since the lesion was associated with the lungs, suggesting the respiratory route as the most probable mode of transmission. Pulmonary granulomatous infection has been reported previously in a -year-old Camargue stallion that had direct contact with infected cattle and shared potentially contaminated pasture [5]. The previous history of this horse and disease status of cograzing individuals was not known.

Genotyping results of the *M. bovis* isolated revealed a spoligopattern (SB0868) and VNTR profile not previously identified in South African wildlife or livestock. The affected horse had been born and was resident in another province, with one of the lowest incidences of bovine tuberculosis in the country, before being relocated after purchase. Based on the time frame between the dates of purchase and presentation for epistaxis, it is highly improbable that the horse had contracted the disease while in the possession of the new owner. Unfortunately, no further historical details were available which could assist in tracing back the origin of the *M. bovis* strain.

*M. bovis* can be transmitted from horses to humans, and a rare case of zoonotic transmission of *M. bovis* associated with the skin of a horse has been described [6]. This report emphasized the importance of public awareness and control of the disease with special attention to those domesticated animals with close contact to humans such as horses. Horses, like cats, dogs and sheep, are considered incidental spill-over hosts and infection is not sustained within the population concerned [19]. In the present case, *M. bovis* was isolated from a respiratory tract sample, signifying that the horse could have been shedding the organism in respiratory secretions. Before laboratory confirmation of the diagnosis, which was delayed due to the prolonged culture time for MTBC, the horse had multiple contacts with other horses and humans, raising the possibility of disease transmission. While ante-mortem diagnosis of *M. bovis* infection is difficult, it is recommended that horses presenting with similar lesions should be kept isolated until laboratory exclusion of mycobacterial infection, preferably using a more rapid diagnostic method such as PCR.

## Conclusion

In conclusion, this report describes the unusual presentation of epistaxis in a horse secondary to pulmonary tuberculosis, and supports that horses may contract and harbor *M. bovis* despite their reported lower susceptibility compared to other domestic animals. The source of this infection is unknown as the *M. bovis* strain has not previously been detected in domestic animals or wildlife in South Africa. The organism was isolated from a BAL sample, indicating that the horse could have been shedding the organism and thereby presenting the risk of forward transmission at the time. Because of the contagious and zoonotic nature of the disease, it is of outmost importance to ensure that measures are taken to prevent transmission of *M. bovis* between potentially infected horses and in-contact persons.

## Footnotes

1 Olympus Medical System Corporation, Tokyo, Japan.

2 Siemens Polydoros 100, Siemens, Forchheim, Germany.

3 Aloka SSD 630, Aloka, Tokyo, Japan.

4 Baytril 10 %, Bayer Animal Health, Isando, South Africa.

5 KI powder, Kyron laboratories, Johannesburg, South Africa.

6 Sonorite A6V, Sonoscape Co. Ltd, China.
